# Learning motivation and environmental support: how first-generation college students achieve success?

**DOI:** 10.3389/fpsyg.2023.1280783

**Published:** 2023-11-30

**Authors:** Xiaojing Li, Weitong Liu, Ke Hu

**Affiliations:** ^1^School of Marxism, Fujian Normal University, Fuzhou, Fujian, China; ^2^School of International Education, Shandong University, Jinan, Shandong, China; ^3^School of Education, Fujian Normal University, Fuzhou, Fujian, China

**Keywords:** first-generation college students, learning motivation, environmental support, learning outcomes, China

## Abstract

**Introduction:**

With the continuous expansion of higher education worldwide, the academic performance of first-generation college students has become an essential topic in the scope of international educational research. This study examines the impact of learning motivation (i.e., intrinsic and extrinsic motivation) and environmental support (i.e., instructional, relational, and campus support) on the academic outcomes of first-generation college students based on the Cultural Mismatch Theory and Self-Determination Theory from both individual and environmental perspectives.

**Methods:**

A two-stage stratified sampling strategy was adopted to guarantee that the samples were representative of the national populations of college students in mainland China. 87418 data were collected from the China College Student Survey (CCSS) 2020, among which 58,864 were first-generation college students. This study primarily employed descriptive statistical analysis and regression analysis methods.

**Results:**

Data analysis revealed that intrinsic learning motivation, relational support, campus support, and academic performance of first-generation college students are significantly lower than those of non-first-generation students. However, this study found no significant differences concerning external learning motivation and teaching support. Regression analysis showed that both learning motivation and environmental support positively predicted learning outcomes, with intrinsic learning motivation having the most substantial influence. Moreover, learning motivation and environmental support interact in jointly promoting the student’s academic success.

**Discussion:**

This study highlighted that the academic development of first-generation college students results from the interplay between individual characteristics and the surrounding environment. Postnatal factors, particularly individual learning motivation, and institutional environment support, play a crucial role in their academic achievements.

## Introduction

As global higher education expands, the study of first-generation college students and their learning issues has become a significant focus in international education research (Ives and Castillo-Montoya, 2020; LeBouef and Dworkin, 2021). Initially defined as college students whose parents have not completed any education beyond the secondary level (Curtin and Cahalan, 2005), the term later evolved to encompass students whose parents had not obtained a bachelor’s degree. This group constitutes a significant portion of the college student population, particularly in countries where higher education is not yet universally accessible, such as China, where over 70% were first-generation college students (Zhang et al., 2016). The expansion of higher education has led to the continued growth of this demographic. Consequently, addressing the learning challenges and educational quality of first-generation college students has become a crucial factor influencing the quality of higher education.

Studies from the United States have found that first-generation college students are more likely to come from lower socioeconomic backgrounds, often belonging to minority groups. Unlike non-first-generation college students, they are more likely to leave college without obtaining a degree (Manzoni and Streib, 2019). Constrained by factors such as limited family, cultural, and social capital, first-generation students face challenges in academic performance, lack of learning motivation, and limited engagement in campus activities (López et al., 2023). Addressing how to help first-generation college students achieve success has become a critical issue in higher education expansion. From the definition of first-generation college students, it is evident that their characteristics are predominantly shaped by their family backgrounds, which are mainly pre-existing and challenging to change (McFadden, 2016). Nevertheless, individuals are malleable, and their development is influenced not only by pre-existing family factors but also by postnatal education and individual subjective agency. Moreover, their efforts can help compensate for innate disadvantages (Museus and Chang, 2021; Acevedo and Lazar, 2022). This flexibility in human development is one of the key reasons why numerous studies focus on the academic experiences of first-generation college students during their educational journey.

Many studies emphasize the causes of academic difficulties among first-generation college students and investigate how external supportive conditions can help them achieve academic success (Watts et al., 2023). Most of this research employs social and cultural capital theories to understand the various inequalities that first-generation college students face in the university environment (Manzoni and Streib, 2019; Glass, 2023). Some studies use dropout and engagement theories to explore the reasons for academic challenges, dropout rates, and low engagement among first-generation college students. They find that family support and campus belongingness can improve retention and academic performance (Roksa and Kinsley, 2019; Museus and Chang, 2021). A few studies have discovered that not all first-generation college students are academically disadvantaged. Although they may lack the cultural capital emphasized in higher education, the cultural environment in which they are raised can also provide certain advantages, such as resilience in facing challenges and cultural diversity (Buie, 2018). This indicates that the factors affecting the academic success of first-generation college students are complex. Therefore, existing research mainly focuses on improving the external environment to facilitate the academic success of first-generation college students but overlooks the significant role of individual intrinsic factors. Understanding the intricate factors influencing first-generation college students’ success is essential. While external conditions play a vital role, the inherent motivation of first-generation students is equally important. This study explores how intrinsic learning motivation and external environmental support affect student academic achievements, considering these two factors as parallel and mutually influential. We situate our study within the Chinese context to address this research objective. In China, students are known to perform well in academic tests yet exhibit low intrinsic motivation, a phenomenon referred to as the “Chinese learner paradox” (Tian and Yu, 2021). This study contributes to reevaluating this phenomenon and offers new perspectives and evidence for research on first-generation college students in different cultural settings.

## Literature review

### Research on first-generation college students

The study on first-generation college students originated from the concerns of experts in the American educational community about the decline in the quality of higher education. Subsequently, the research expanded to the domain of educational equity since first-generation college students are widely regarded as relatively disadvantaged in terms of cultural capital and entail the phenomenon of social intergenerational mobility (Williams and Ferrari, 2015). Numerous studies have indicated that compared to non-first-generation college students, most first-generation college students come from rural areas with lower socioeconomic status, exhibit academic underperformance, and experience higher dropout rates (Gibbons and Woodside, 2014). Consequently, previous research has primarily focused on the academic performance of first-generation college students and the intergenerational transmission of education between them and their parents, such as their access to educational opportunities, academic achievements, and retention rates (Riehl, 1994; Pratt et al., 2019).

Higher education has been transitioning from massification to universalization in China recently. At the same time, this transition has also brought about educational challenges similar to those during the transformation period of higher education in the United States. Consequently, an increasing number of studies have started focusing on the educational issues faced by first-generation college students. First, research has revealed that the proportion of first-generation college students in elite universities in China is gradually decreasing, with only 59.3% gaining admission to top-tier universities (Zhao et al., 2014). Second, first-generation college students are disadvantaged regarding admission type, academic performance, and occupation choices. Specifically, first-generation college students exhibit lower academic engagement on campus (Pratt et al., 2019; Marco-Bujosa et al., 2023). Despite various learning problems and challenges, first-generation college students can still overcome difficulties and obtain bachelor’s degrees (Buie, 2018), partly due to their inherent strengths. As research on first-generation college students delves deeper, some scholars in China have also started to focus on how the advantages exhibited by this group help them overcome difficulties. It has been found that first-generation college students demonstrate strong resilience and perseverance in pursuing academic success due to their cultural qualities and characteristics (Yu and Han, 2018; Chang et al., 2020). In a word, researchers hold two contrasting views on the learning issues and outcomes of first-generation college students.

The emergence of these two contrasting perspectives may be related to the social and cultural environment in which first-generation college students are situated. First-generation college students represent a diverse and heterogeneous group (Tian and Yu, 2021), encompassing factors such as gender, nationality, urban–rural background, social class, and so on, leading to significant internal differences. Furthermore, their experiences are also influenced by the cultural context of their country. However, first-generation college students generally share the common characteristic of coming from families with lower socioeconomic status. For example, in the United States, first-generation college students come from diverse backgrounds, have higher dropout rates, and experience less urban–rural disparity. In contrast, first-generation college students in China have a lower college dropout rate but face significant urban–rural inequality and uneven economic development. It is evident that the current landscape of first-generation college students exhibits a diversity of characteristics, necessitating a consideration of both their internal structures and the external cultural environmental factors.

### Research on the impact of innate factors on the academic success of first-generation college students

Innate factors, which are economic and social capital from parents, play a fundamental role, though not the sole determinant, in first-generation students’ academic performance. From an individual perspective, the lower socioeconomic status and limited cultural capital in the families of first-generation students significantly affect their academic achievements. Compared to non-first-generation students, first-generation students face more significant challenges in accessing higher education opportunities, adapting to university-level studies, and managing to graduate due to their lack of essential cultural capital (Dumais and Ward, 2010). Research conducted by Chinese scholars also reveals that family cultural norms, parental educational support, and educational expectations have a notable impact on students’ academic performances. In addition to family cultural capital, family economic and social capital also influence the provision of learning resources and the academic performance of first-generation college students. On one hand, economic difficulties within families may hinder first-generation students from completing their education (Wang and Nuru, 2017). Specifically, they may lack sufficient financial support to participate in high-impact educational activities such as studying abroad, engaging in scientific research, or pursuing a second degree, all of which contribute significantly to students’ academic development (Zhang et al., 2017). On the other hand, family social capital also significantly affects first-generation students’ academic performance. Research has discovered that these students lack sufficient social network support to help them smoothly navigate their educational journey. They face substantial challenges in pursuing further education, applying for scholarships, and seeking financial assistance (Hébert, 2018).

In addition to the direct influence on academic performance, innate factors also affect their academic achievements through mediating factors such as learning motivation, emotional support, etc. Firstly, active parental guidance in learning, involvement in college planning, and stimulating a child’s desire to learn can enhance the autonomy and motivation of first-generation college students to pursue higher education (Mitchall and Jaeger, 2018). This phenomenon is quite common in the Chinese cultural context, where parents who have not attended college often transfer their educational expectations to their first-generation college-student children, creating favorable conditions for their learning and encouraging them to repay their parents through academic success and social mobility (Chen and Kang, 2018). However, there are also families that, due to a lack of advanced educational awareness or financial ability, uphold the notion that education is useless, resulting in lowered academic expectations, restricted choices, and weakened learning motivation among first-generation college students. Secondly, research indicates that emotional support from the family helps first-generation college students adapt to the college environment and achieve academic success (Roksa and Kinsley, 2019). On the other hand, a gap between family members who have not attended college and academic learning might lead to less emotional support, resulting in some issues like feelings of inferiority, isolation, and low self-efficacy for first-generation college students (Covarrubias et al., 2015; Harackiewicz and Priniski, 2018; Page et al., 2019).

### Research on the impact of environmental factors on the academic success of first-generation college students

Although inherent background factors that students possess are relatively stable and difficult to change, students can still enhance their academic achievements through individual efforts. Environmental support from universities can facilitate the academic success of first-generation college students. Empirical studies have also demonstrated the significant impact of a supportive university environment on students’ capacity development (Bao, 2020). For example, research has found a positive correlation between the level of university support environment and students’ GPA. The university support environment also substantially impacts students’ general capabilities, practical skills, and personal values (Kitchen et al., 2021). The university environment directly influences students’ academic performance and indirectly affects learning outcomes by influencing individual-level learning engagement (such as individual effort quality and teacher-student relationships). Among various aspects of the university support environment, teaching support strongly influences students’ learning engagement and outcomes. When teachers provide timely guidance and support, students tend to exhibit a higher level of learning interest, leading to academic success (Tao et al., 2022).

Additionally, the teacher-student relationship, as an essential dimension of the university environment, significantly influences students’ learning engagement and outcomes. Interpersonal relationships such as peer interaction and cooperation, as well as teacher-student interaction and communication, impact students’ deep learning mechanisms, consequently affecting academic achievements (Hagenauer et al., 2023). Challenging peer relationships encountered by first-generation college students, imposed by their socioeconomic status, make it hard to fit into higher education environments smoothly, which hinders their enthusiasm to interact with others (Rubin, 2012). Furthermore, in elite universities, the sense of exclusion experienced by first-generation college students is even more pronounced.

The mechanism of the impact of environmental factors on the learning outcomes of first-generation college students is often explained by cultural capital theory and cultural mismatch theory. Cultural capital theory posits that family cultural capital is resistant to change, but school cultural capital can be strategically allocated to assist first-generation college students in achieving success. Factors including the integration of campus culture, normative capital, and the symbiotic relationship between educational institutions and families play a crucial role in shaping first-generation college students’ academic and career progression (Garriott, 2020). The college experience provides a means for first-generation students to acquire compensatory cultural and social capital. Specifically, the university environment can act as a compensatory factor, given the relative scarcity of family cultural capital among first-generation students compared to their non-first-generation counterparts (Pascarella et al., 2004). Therefore, a supportive institutional environment becomes particularly vital in the upbringing of first-generation college students. Cultural mismatch theory asserts that the primary reason for underperformance among the first-generation college student group is the incongruence between the mutual interdependence norms of this group and the prevalent independent norms in the university setting. This mismatch reduces the first-generation students’ ability to address challenges and seek assistance, thereby impeding their effective utilization of support from both society and universities (Chang et al., 2020).

### Analytical framework

The Joint Committee on Standards for Educational Evaluation in the United States defines student learning outcomes as the “knowledge and comprehension(cognition), practical skills (skill), attitudes and values (affection), and individual behavior,” which students should attain upon completing courses and obtaining degrees (Arlen, 2003). Kuh argues that learning gain refers to the ability of students to demonstrate the knowledge, skills, and values they have acquired after completing a series of courses or a developmental plan; this serves as a measure of student development (Kuh and Hu, 2001). Student academic success encompasses objective grade improvement and enhancement of knowledge, skills, and values through on-campus learning.

Social Cognitive Theory, developed by Bandura (1986), posits that learning is affected by individual, behavioral, and environmental factors. In higher education research, student academic success is the outcome of the interaction between individual learning behaviors and the institutional environment. The theory of Student Engagement proposed by Kuh (2009) also emphasizes that student engagement includes both the time and experiences students invest in learning activities and the overall environment created by the institution to facilitate student learning. By integrating core perspectives from identity motivation theory, interindividual cultural theory, and person-environment fit theory, the cultural mismatch theory focuses on the interrelationship between individuals and the cultural environment, suggesting that the university environment plays a crucial role in influencing individuals’ learning motivation, behavior, and academic performance. Cultural norms within the university environment have varying effects on students from different backgrounds, with the success of college students depending on the alignment between their self-pattern and university culture (Stephens et al., 2019). Non-first-generation college students tend to have a higher alignment with cultural norms.

In contrast, the mismatch between first-generation college students and the dominant cultural model in higher education subjects them to more psychological challenges. While the cultural mismatch theory explains the reasons for the academic challenges faced by first-generation college students, it neglects the significant role of individual subjective agency (Phillips et al., 2020). The self-determination theory supplements this aspect by emphasizing the importance of personal intrinsic motivation and focusing on the extent to which human behavior is voluntary or self-determined (Gutierrez-Serrano et al., 2022).

**Figure 1:**
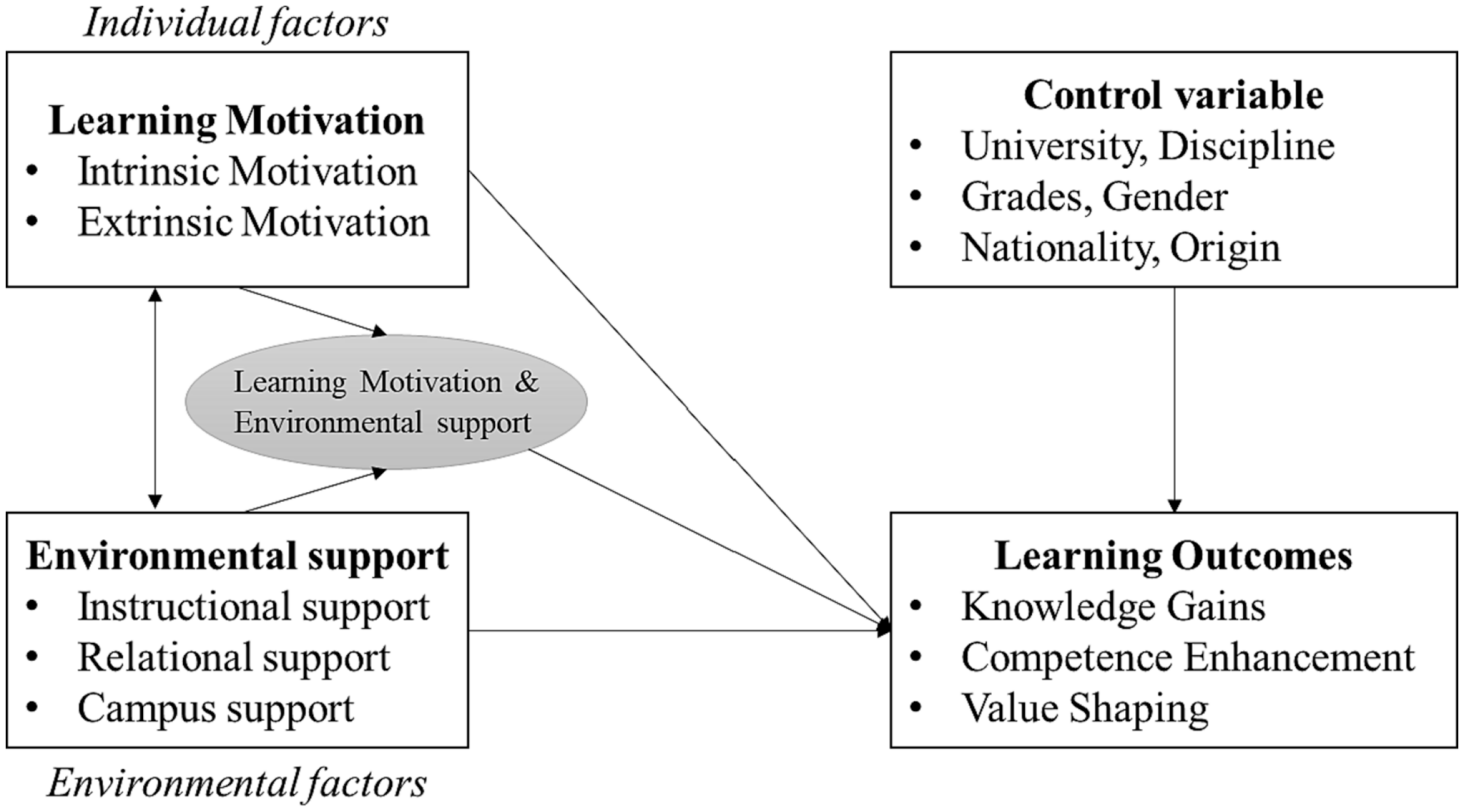
Conceptual model.

Therefore, from a comprehensive perspective, the academic success of first-generation college students depends on whether their subjective agency and learning motivation are stimulated and whether sufficient environmental support is provided. In fact, existing literature has suggested integrating both individual and environmental factors that influence the academic development of first-generation college students, particularly the role of postnatal individual and environmental factors. Focusing on first-generation college students in China, this study examines the integrated influence of individual and environmental factors on their academic outcomes. Figure 1 illustrates the conceptual model of this study. By drawing upon the theoretical framework and existing literature, this study primarily discusses the impact of individual factors (learning motivation) and environmental factors (environmental support) on students’ learning outcomes, resulting in research hypotheses H1 and H2. It’s worth noting that individual and environmental factors interact with each other, influencing students’ learning outcomes. Consequently, the interaction between these two factors is discussed, leading to research hypothesis H3.

*H1*: Learning motivation positively predicts learning outcomes of first-generation college students;

*H2*: Environmental support positively predicts the learning outcomes of first-generation college students;

*H3*: An interaction between learning motivation and environmental support positively predicts students’ learning outcomes.

## Methodology

### Instruments

The data used in this study are sourced from the China College Student Survey (CCSS) 2020. The CCSS project has been running for over a decade since its nationwide initiation in 2009. The survey questionnaire has undergone repeated revisions and improvements, demonstrating strong measurement reliability and validity. It has been widely employed in research areas related to students’ learning engagement, satisfaction, and factors influencing their educational achievements. The CCSS questionnaire primarily captures students’ learning experiences and educational outcomes during their academic terms. The questionnaire is divided into two parts: Part A mainly reflects students’ learning experiences during the period, with question options using a 4-point or 7-point Likert scale. Part B includes demographic characteristics of students, family backgrounds, and other related information.

### Sample technique and procedures

A two-stage stratified sampling strategy was adopted to guarantee that the samples were representative of the national populations of college students in mainland China. First, institutions were selected with a stratified random sampling strategy by institution type and geographic area. Among the 32 universities, five were designated as World-Class Universities, nine as universities with World-Class Discipline, and 18 as other undergraduate institutions. Second, participants from each university were stratified based on grade, gender, and discipline, with a sample size of 400 to 800 students selected for each grade. In total, 136,877 questionnaires were distributed for the study, of which 107,726 were collected, resulting in a response rate of 78.42%. After excluding invalid and non-randomly sampled questionnaires, as well as cases with missing data, the final sample used in this study consisted of 87,418 valid responses. The distribution of the sample is shown in Table 1.

**Table 1:** Sample distribution.

Terms	Categories	Sample size	Percentage (%)
University	World-Class University	13,810	15.80
	University with World-Class Discipline	27,414	31.36
	Others	46,194	52.48
Discipline	Humanities	9,636	11.02
	Social sciences	23,380	26.75
	Natural sciences	5,907	6.76
	Engineering	42,750	48.91
	Biomedical sciences	5,736	6.56
Grades	First year	25,280	28.92
	Second year	23,191	26.53
	Third year	23,296	26.65
	Fourth year	15,651	17.90
Gender	Male	45,784	52.37
	Female	41.634	47.63
Nationality	Han	78,382	89.66
	Minority	9,036	10.34
Origin	Urban areas	67,984	77.77
	Rural areas	19,434	22.23
First-generation College students	No	28,554	32.66
	Yes	58,864	67.34

According to the construction plan for Chinese universities, Chinese universities are categorized into World-Class Universities, universities with World-Class Discipline, and others.

### Variables

The dependent variable in this study is the self-reported learning outcomes of college students, that is, the perceived degree of improvement in knowledge, capability, values, and other aspects reported by the students. Specifically, it includes three dimensions: knowledge gains, capability enhancement, and value shaping. Knowledge gains consist of four items, capability enhancement eight items, and value shaping three items; the details of the questionnaire are attached by complemental materials.

In this study, we conducted a confirmatory factor analysis (CFA) to assess the structural validity of the learning outcomes using Mplus 7.0. CFA is widely employed in psychometrics to confirm the underlying factor structure of a set of observed variables. This analysis helps verify the extent to which the observed variables relate to the hypothesized constructs, allowing us to examine the validity of the measurement model. In our study, the CFA aimed to test the underlying structure of the learning outcomes to ensure that the selected measurement items appropriately represented the latent constructs. The following fit indices were employed to evaluate the model’s goodness-of-fit: *x^2^* = 86830.59, *df* = 882, *CFI* = 0.93, *TLI* = 0.92, *RMSEA* = 0.05. These indices indicate that the measurement model fits the data well. Specifically, the Comparative Fit Index (CFI) and Tucker-Lewis Index (TLI) values above 0.90, along with the Root Mean Square Error of Approximation (RMSEA) value below 0.08, are indicative of a satisfactory fit.

Furthermore, the factor loadings of the measurement items for the learning outcomes variable ranged from 0.75 to 0.85. These factor loadings represent the strength of the relationship between each observed item and the underlying construct. An item with a factor loading exceeding 0.70 indicates a strong indicator of the respective construct. We performed an internal consistency analysis to assess the reliability of the measurement items within each dimension. The analysis yielded Cronbach’s alpha coefficients of 0.86, 0.94, and 0.86 for knowledge gains, capability enhancement, and value shaping, respectively. These coefficients significantly exceed the acceptable threshold of 0.70, demonstrating a high level of scale reliability and indicating that the measurement items consistently measure the intended constructs.

The core independent variables in this study are learning motivation (individual) and environmental support (environment). Understanding motivation is often approached from a psychological perspective, referring to the driving tendency that initiates and sustains students’ learning behavior and helps them achieve specific academic goals. In Chinese culture, students’ learning motivation is based on an interdependent self-concept, reflecting individuals’ relationships with others, collectives, and society. It emphasizes both personal self-development and societal expectations. Research has categorized it into intrinsic and extrinsic motivation types (Zhang et al., 2021). In this study, learning motivation is divided into intrinsic and extrinsic motivation. Intrinsic motivation emphasizes self-fulfillment, while extrinsic motivation emphasizes meeting social expectations. The learning motivation construct consists of eight items, with a Cronbach’s alpha coefficient of 0.77. Intrinsic motivation comprises six items with a Cronbach’s alpha of 0.81, while extrinsic motivation consists of two items with a Cronbach’s alpha of 0.50. Environmental support is based on the three basic psychological needs of self-determination theory and is divided into instructional support, relational support, and campus support. The environmental support construct includes 18 items, with a Cronbach’s alpha coefficient of 0.92. Instructional support refers to teachers providing timely guidance and feedback for student learning, comprising five items with a Cronbach’s alpha of 0.91. Relational support represents the quality of relationships between students, teachers, peers, and administrative staff, consisting of four items with a Cronbach’s alpha of 0.81. Campus support refers to the support conditions provided by the university for students’ overall development, including nine items with a Cronbach’s alpha of 0.94.

Control variables include individual background variables such as gender and origin (rural or urban areas), as well as institutional background variables such as university categories and disciplines. Universities in this study are categorized as World-Class universities, universities with World-Class Discipline, and other undergraduate institutions. Disciplines are divided into five categories: humanities, social sciences, natural sciences, engineering, and biomedical sciences. As the CCSS questionnaire relies on student self-reporting, and self-reporting can be influenced by social desirability bias (Guo et al., 2018), this study also includes social desirability as a control variable in the analysis.

### Analysis method

This study primarily employed descriptive statistical analysis and regression analysis methods. Initially, we analyzed the learning motivation, environmental support, and academic performance of first-generation university students across different types of institutions, genders, and disciplines. Subsequently, we utilized regression analysis to investigate the influence of learning motivation and environmental support on academic outcomes, with academic performance as the dependent variable. We specifically focused on learning motivation and environmental support as the primary independent variables while simultaneously adding some control variables. Both the aforementioned descriptive statistical analysis and multiple regression analysis were conducted using Stata 13. Given the structural differences between the sample and the population, sample weighting methods were employed to correct potential biases during the analysis, ensuring a more detailed explanation of the methodology.

## Results

### Demographics of first-generation college students

**Figure 2:**
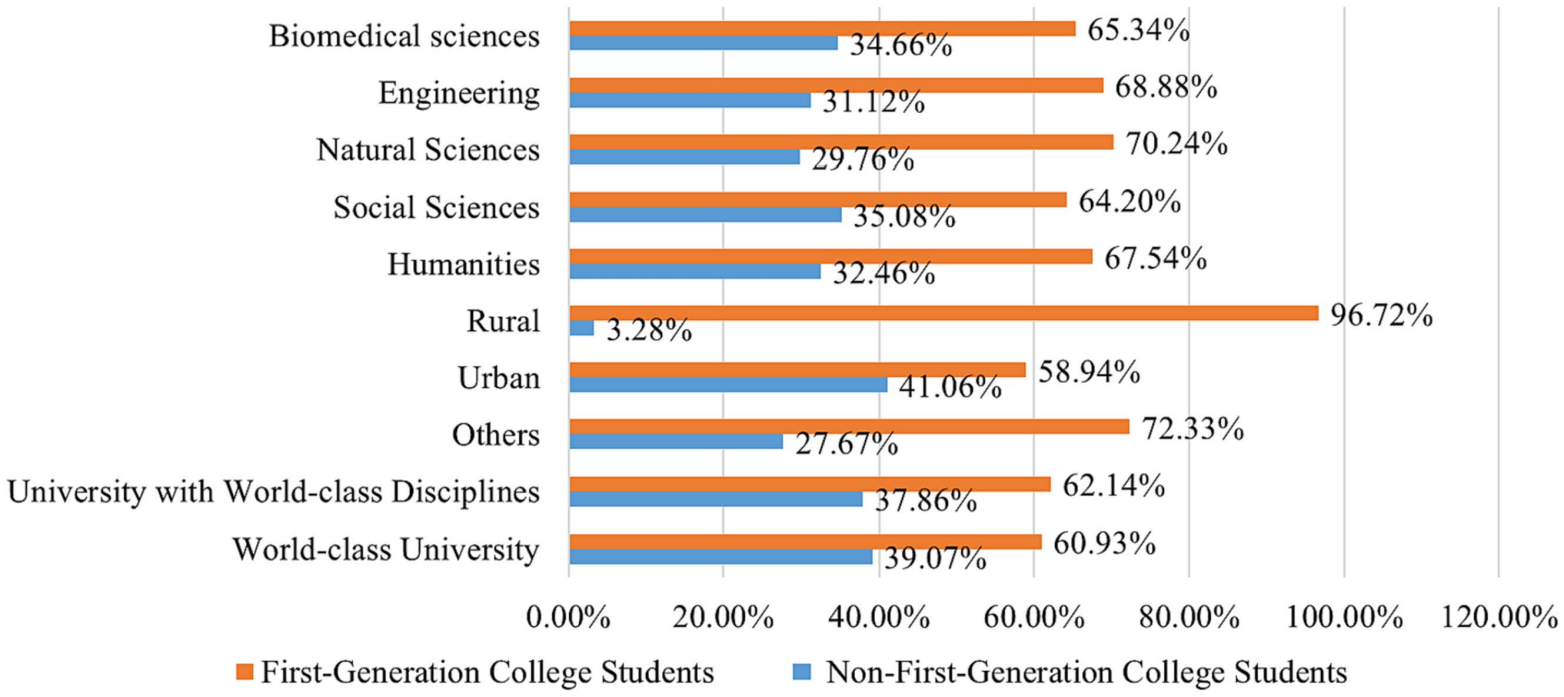
Distribution of first-generation and non-first-generation college students by university category, discipline, and origin.

Figure 2 displays the distribution of first-generation and non-first-generation college students in China based on university category, discipline, and origin. The results of the chi-square test indicated significant differences in the distribution of first-generation college students in terms of university type (*χ^2^* = 1.1e+03, *p* < 0.001), academic discipline (*χ^2^* = 184.16, *p* < 0.001), and hometown (*χ^2^* = 9.8e+03, *p* < 0.001). As depicted in Figure 2, the proportion of first-generation college students is higher than that of non-first-generation students in terms of university categories, disciplines, and origin. This suggests that first-generation college students remain a predominant group in Chinese universities. Among them, the proportion of first-generation college students attending the World-Class university (60.93%) and universities with the World-Class Discipline (62.14%) is significantly lower than that of other universities (72.33%). Furthermore, first-generation college students from rural areas account for as much as 96.72%, and those studying STEM (science, technology, engineering, and mathematics) fields exceed those in other disciplines. This indicates that first-generation college students are less represented in elite universities and urban areas and tend to pursue applied fields.

### Learning motivation, environmental support, and learning outcomes of first-generation college students

Table 2 displays the performance disparities among first-generation and non-first-generation college students concerning learning outcomes, learning motivation, and environmental support. The results indicated notable variations between the two groups concerning learning outcomes (*t* = 24.56, *p* < 0.001), learning motivation (*t* = 8.43, *p* < 0.001), and environmental support (*t* = 6.33, *p* < 0.001).

**Table 2:** Performance of First-Generation College Students in learning motivation, environmental support, and learning outcomes.

	First-generation college students		Non-first-generation college students		
	Mean	Std.	Mean	Std	*t*
Learning outcomes	59.62	19.44	63.10	20.18	24.56***
Knowledge	60.1	22.24	63.53	23.09	21.09***
Capability	58.52	20.56	62.57	21.22	27.04***
Value	62.39	23.3	64.68	24.73	13.53***
Learning Motivation	67.91	13.71	68.76	14.48	8.43***
Intrinsic motivation	67.37	15.34	68.61	16.46	10.91***
Extrinsic motivation	69.25	18.7	69.13	20.08	−0.86
Environmental support	71.74	13.68	72.38	14.70	6.33***
Relational support	66.65	17.99	66.8	19.48	8.74***
Instructional support	73.37	18.31	74.54	18.67	1.12
Campus support	73.76	18.43	74.52	16.7	5.87***

***p < 0.001.

When considering learning outcomes, it is noteworthy that self-reported learning achievements among first-generation college students (*mean* = 59.62) exhibited a significant difference from those of non-first-generation students (*mean* = 63.10). This disparity was consistently reflected in knowledge gains (*t* = 21.09, *p* < 0.001), capability enhancement (*t* = 27.04, *p* < 0.001), and value shaping (*t* = 13.53, *p* < 0.001), underscoring the academic performance gap between first-generation college students and their non-first-generation peers.

Regarding learning motivation, first-generation college students (*mean* = 67.91) exhibited lower levels of intrinsic motivation compared to non-first-generation students (*mean* = 68.76; *t* = 8.43, *p* < 0.001), but there was no significant difference in extrinsic motivation (*t* = −0.86, *p* = 0.39). This suggested that first-generation and non-first-generation college students differed in aspects such as learning interests, self-challenge, and enjoyment of learning. Still, there was no difference in learning for meeting external societal expectations.

In terms of environmental support, first-generation college students (*mean* = 71.74) perceived lower levels of support compared to non-first-generation students (*mean* = 72.38; *t* = 6.33, *p* < 0.001). Among various forms of support, first-generation students perceived the highest level of campus support (*mean* = 73.76) and the lowest level of relational support (*mean* = 60.65). Significant differences existed between first-generation and non-first-generation students in terms of relational support (*t* = 8.74, *p* < 0.001) and campus support (*t* = 5.87, *p* < 0.001), while no significant difference was detected in instructional support (*t* = 8.43, *p* = 0.26). This indicated that the perceived teacher-student relationships, peer relationships, and campus support provided by institutions differed between the two groups, but there was no noticeable difference in the feedback and support provided by teachers.

### Influence of learning motivation and environmental support on learning outcomes

In order to investigate the explanatory power and relative significance of learning motivation and environmental support on learning outcomes, regression analysis was performed on a sample of 58,869 first-generation college students. The regression results are presented in Table 3. The regression results of Model 1 indicated that both learning motivation and environmental support had positive predictive effects on student learning outcomes. The predictive impact of learning motivation (*β* = 0.494, *p* < 0.001) on learning outcomes was higher than that of environmental support (*β* = 0.429, *p* < 0.001), as Table 3 Model 1 shows, suggesting that learning motivation has a more significant impact on student learning outcomes. Concerning the correlation between learning motivation and environmental support, an interaction term between learning motivation and environmental support is added in Model 2. Further results demonstrated that the interaction between learning motivation and environmental support had a positive predictive effect on learning outcomes (*β* = 0.00483, *p* < 0.001). This suggested that learning motivation and environmental support could interact to enhance the overall quality of student learning and development to a greater extent.

**Table 3:** The impact of learning motivation and environmental support on learning outcomes of first-generation college students.

Variables	Model 1	Model 2	Model 3	Model 4	Model 5
	Learning outcomes	Learning outcomes	Knowledge gains	Capability enhancement	Value shaping
Learning motivation	0.494***	0.139***			
	−0.008	−0.036			
Intrinsic motivation			0.449***	0.390***	0.485***
			−0.011	−0.010	−0.017
Extrinsic motivation			0.0864***	0.0954***	0.0931***
			−0.012	−0.007	−0.011
Environmental support	0.429***	0.0933*			
	−0.009	−0.036			
Relational support			0.123***	0.146***	0.134***
			−0.009	−0.008	−0.012
Instructional support			0.143***	0.119***	0.104***
			−0.009	−0.012	−0.011
Campus support			0.154***	0.160***	0.163***
			−0.018	−0.018	−0.010
Learning motivation*		0.00483***			
Environmental support		0.000			
Desirability	0.155***	0.154***	0.124***	0.155***	0.128***
	−0.006	−0.006	−0.004	−0.007	−0.008
Social sciences	2.104***	2.090***	1.508*	2.867***	1.828**
	−0.380	−0.362	−0.600	−0.365	−0.565
Sciences	1.704***	1.659***	1.648*	2.578***	1.088*
	−0.291	−0.282	−0.614	−0.366	−0.476
Engineering	3.148***	3.100***	3.674***	4.201***	1.787***
	−0.279	−0.278	−0.454	−0.312	−0.447
Biomedical sciences	2.344***	2.306***	3.555***	2.127*	3.327***
	−0.645	−0.625	−0.616	−0.840	−0.509
Other nationalities	−1.253**	−1.226**	−1.437***	−1.138*	−1.339*
	−0.429	−0.413	−0.341	−0.467	−0.597
Rural	−2.497***	−2.452***	−2.243***	−2.936***	−1.301***
	−0.270	−0.272	−0.302	−0.321	−0.330
University with World-Class Discipline	−1.849*	−1.897*	−2.913	−1.958*	−1.565
	−0.817	−0.835	−1.456	−0.859	−0.817
Other University	−1.174	−1.201	−1.781	−1.408*	−0.236
	−0.593	−0.597	−0.989	−0.615	−0.724
Second year	1.601***	1.564***	1.676***	1.710***	0.831
	−0.317	−0.312	−0.387	−0.364	−0.421
Third year	2.958***	2.905***	2.520***	3.156***	3.199***
	−0.272	−0.274	−0.191	−0.301	−0.485
Fourth year	4.393***	4.287***	3.760***	4.758***	3.688***
	−0.404	−0.400	−0.452	−0.412	−0.391
Constant	−15.64***	8.646**	−14.79***	−16.37***	−14.99***
	−1.169	−2.754	−1.657	−1.366	−1.025
Sample size	58859.000	58859.000	58859.000	58859.000	58859.000
r2	0.452	0.456	0.358	0.406	0.350
r2_a	0.452	0.456	0.358	0.406	0.350

*p < 0.05, **p < 0.01, ***p < 0.001.

The regression results of Model 3, Model 4, and Model 5 indicated that first-generation college students’ intrinsic and extrinsic learning motivation positively affected various aspects of learning outcomes, including knowledge gains, capability enhancement, and value shaping. The impacts of intrinsic learning motivation on knowledge gains (*β* = 0.449, *p* < 0.001), capability enhancement (*β* = 0.390, *p* < 0.001), and value shaping (*β* = 0.485, *p* < 0.001) were all greater than that of extrinsic learning motivation (*β* = 0.086, *p* < 0.001; *β* = 0.095, *p* < 0.001; *β* = 0.093, *p* < 0.001). Among these, intrinsic learning motivation had the greatest impact on value shaping, followed by knowledge acquisition and capability enhancement. This suggests that intrinsic learning motivation was a crucial factor influencing students’ academic success. In terms of environmental support, relational support, instructional support, and campus support all had positive predictive effects on students’ knowledge acquisition, capability enhancement, and value shaping. Among them, relational support had the most significant predictive effect on capability enhancement (*β* = 0.146, *p* < 0.001), instructional support had the most significant predictive effect on knowledge gains (*β* = 0.143, *p* < 0.001), and campus support had the most significant predictive effect on value shaping. This suggested that instructional support helped students acquire knowledge, relational support contributed to the development of students’ overall abilities, and the campus environment played an important role in shaping students’ values.

From the perspective of student background characteristics, university category, origin, discipline, and grade level all had significant predictive effects on student learning outcomes among first-generation college students. Compared to students from World-Class universities, those from universities with Word-Class Discipline and other universities exhibited lower levels of knowledge acquisition, capability enhancement, and value shaping. First-generation college students from rural areas had lower academic performance than their urban counterparts (*β* = −2.452, *p* < 0.001, based on urban area). In terms of disciplines, first-generation college students studying engineering achieved higher academic outcomes than those in other disciplines (*β* = 3.100, *p* < 0.001, based on Humanities). Additionally, senior students in their higher grades achieved higher academic outcomes than junior students among first-generation college students (*β* = 1.564, *p* < 0.001 of the second year; *β* = 2.905, *p* < 0.001 of the third year; *β* = 4.287, *p* < 0.001 of the fourth year based on first-year students). When considering nationality, Han first-generation college students performed better in academic achievements compared to other nationalities (*β* = −1.226, *p* < 0.005, based on Han students).

## Discussion

Firstly, enhancing the academic success of first-generation college students is imperative for improving the quality of higher education. During the phase of higher education massification, with the expansion of higher education and increased opportunities for university enrollment, the number of first-generation college students reached a considerable level for a certain period. As higher education transitions into a stage of universalization, the population of first-generation college students will gradually decrease. By 2020, the proportion of first-generation college students in China had already declined to 67.34%, while in the United States, it had reduced to 56% (NASPA, 2020). However, the reduction in numbers does not imply that research into the academic challenges faced by this group is no longer significant. The diverse composition of first-generation college students calls for heightened attention to their academic challenges (Glass, 2023). The data analysis in this study reveals that first-generation college students in China continue to constitute the majority of university students, although this group exhibits internal diversity. However, their representation in elite universities is gradually decreasing (Bao, 2021), giving rise to issues related to educational inequality and increased risks associated with higher education investments. The context of Chinese culture influences Chinese first-generation college students. Phrases like “noble person from a humble family” and “small-town swot” reflect the unique educational culture of rural Chinese society. On one hand, this suggests that first-generation college students predominantly come from rural areas and are less likely to get rid of the negative impacts of humble origins on academic performance (Zhang et al., 2017). On the other hand, it also signifies the studious and hardworking qualities of Chinese first-generation college students who strive to overcome social barriers. This has prompted scholars to advocate for studying the advantages of first-generation college students while considering their cultural context.

Secondly, innate factors merely provide the groundwork for the development of first-generation college students, while postnatal factors are the pivotal influencers in determining their academic success or challenges. The outcomes of this research underscore that individual learning motivation and environmental support have a positive predictive impact on students’ academic development. Unlike innate attributes, which are not easily altered across individuals’ development, postnatal factors, both stimulation of individual subjective initiative and the creation of conditions for environmental support can make a difference in learning outcomes. Intrinsic learning motivation consistently stimulates students’ academic performance and work achievements (Froiland and Worrell, 2016). The self-determination theory posits that individuals can actively engage, proactively absorb information and behavioral norms, integrate within societal groups, and enhance themselves. While first-generation college students may be constrained by the limitations of their innate cultural environment, they can counteract this through postnatal efforts, employing proactive strategies and utilizing the cultural wealth within their community to address challenges encountered during their development (Almeida et al., 2021; LeBouef, 2023). Chinese first-generation college students are characterized by their diligence, perseverance, and resilience. Activating their inherent qualities and developmental potential is a practical approach to compensate for the disadvantage of lacking innate cultural capital.

Thirdly, the academic success of first-generation college students results from the interplay between individual and environmental factors, and integrating these factors enhances students’ learning outcomes. The data analysis in this study also indicates that both personal learning motivation and institutional environmental support influence the learning outcomes of first-generation college students. There exists a significant interaction between individual learning motivation and the institutional environment. Specifically, a better institutional support environment and a more robust personal learning motivation will result in more remarkable academic achievements for students. Our findings are consistent with Person-environment Theory, which emphasizes the interaction between individual or group attributes and the university environment. For first-generation college students, learning and development are characterized by the simultaneous operation of individual and social variables, and they are co-constructed through the bidirectional interaction between the university context (environment) and learning habits (individuals) (Rocconi et al., 2020; LeBouef and Dworkin, 2021). The data analysis from this study further reveals that different types of environmental support have varying effects on students’ knowledge gains, capability enhancement, and value shaping. Teacher feedback and guidance effectively enhance students’ knowledge acquisition, while campus interpersonal relationships support the development of students’ abilities. The campus cultural environment plays a crucial role in shaping students’ values. This finding underscores the necessity of providing appropriate environmental support based on the needs of first-generation college students’ development. Consistently, Sanford, a prominent figure in the theory of college student development in the United States, suggests that student development emerges from the interaction between individuals and their environment (Evans et al., 2009). To enhance the success of college students, it is imperative to strike a balance between establishing a supportive environment and introducing challenges.

## Conclusion and implications

In sum, first-generation college students have been a prominent topic in education. This study, conducted within the context of China, examines how students’ learning motivation and university environment support impact the academic success of first-generation college students from a developmental perspective. The research highlights that the academic development of first-generation college students results from the interplay between individual characteristics and their surrounding environment. Postnatal factors, particularly individual learning motivation, and institutional environment support, play a crucial role in their academic achievements.

However, several limitations in this study warrant consideration: Firstly, the factors influencing the academic success of first-generation college students are multifaceted. The interplay and mechanisms between innate characteristics and postnatal factors need further investigation. Understanding the complex relationship and interactions between these factors could provide a more comprehensive understanding of the dynamics at play. Secondly, this study is limited to the context of China. Engaging in dialog with research findings on first-generation college students in Western contexts is essential to facilitate a cross-cultural understanding of this phenomenon.

In future research, there are a few avenues to explore. Firstly, conducting cross-cultural studies could further analyze the academic performance of first-generation college students from different cultural backgrounds. Adopting a developmental perspective and exploring individual agency, these studies could delve into first-generation students’ postnatal influences and potential advantages. Secondly, employing a mixed-methods approach could be beneficial. By integrating the analysis of large-scale survey data with qualitative research methods such as case studies, ethnography, and phenomenology, researchers could gain deeper insights into the experiences of first-generation college students at a micro level.

This study confirms the importance of learning motivation and environmental support in the academic development of first-generation college students, where learning motivation and environmental support interact and jointly promote the academic success of these students. External environmental factors influence internal individual factors, with environmental support igniting students’ learning motivation. The interaction between these factors enhances students’ learning outcomes. Based on the results of this study, it is recommended that higher education institutions facilitate the academic success of first-generation college students in two main ways. Firstly, by assisting first-generation students in achieving self-driven success. Igniting intrinsic learning motivation, particularly internal motivation, is critical to promoting academic success. Higher education institutions should offer equal admission opportunities to students, enabling them to access higher education through their efforts. Institutions should also provide the necessary psychological support to help first-generation students discover their cultural strengths, boost their self-confidence, and enhance their autonomy in learning. Moreover, creating an inclusive cultural environment is necessary to prevent identity discrimination from cultural mismatches. Secondly, by creating a conducive environment for their external-driven success. Environmental support is a critical factor that drives first-generation students externally. Higher education institutions should offer teaching support to help them complete their studies. Educators should pay attention to the academic development of first-generation students, encouraging their participation in various educational activities. Additionally, institutions should establish spaces and opportunities for teacher-student interactions, enhance the quality of student-faculty relationships, and provide diverse and compensatory campus cultural activities to address the lack of cultural capital among these students.

## Data availability statement

The data analyzed in this study is subject to the following licenses/restrictions: Data from Tsinghua University “China College Student Survey,” but this is not a public database. Requests to access these datasets should be directed to hukeflame99@126.com.

## Ethics statement

Ethical review and approval was not required for the study on human participants in accordance with the local legislation and institutional requirements. Written informed consent from the patients/ participants or patients/participants' legal guardian/next of kin was not required to participate in this study in accordance with the national legislation and the institutional requirements.

## Author contributions

XL: Conceptualization, Data curation, Formal analysis, Methodology, Project administration, Writing – original draft, Writing – review & editing. WL: Data curation, Formal analysis, Methodology, Software, Writing – review & editing. KH: Data curation, Investigation, Project administration, Validation, Writing – original draft, Writing – review & editing.

## References

[ref1] Acevedo E. Lazar A. J. (2022). Active learning and interpersonal skills development among first-generation college students. Int. Stud. Perspect. 23, 249–270. doi: 10.1093/isp/ekab010

[ref2] Almeida D. J. Byrne A. M. Smith R. M. Ruiz S. (2021). How relevant is grit? The importance of social capital in first-generation college students’ academic success. J. Coll. Stud. Retent.: Res. Theory Pract. 23, 539–559. doi: 10.1177/1521025119854688

[ref3] Arlen R. G. (2003). The student evaluation standards: How to improve evaluations of students. Cali-fornia: Educational Policy Leadership Institute.

[ref4] Bandura A. (1986). Social foundations of thought and action. Englewood Cliffs, NJ, Prentice Hall.

[ref5] Bao Z. M. (2020). The current situation of university learning environment and its influence on undergraduate Students' competence development. Jiangsu High. Educ. 3, 15–22. doi: 10.13236/j.cnki.jshe.2020.03.003

[ref6] Bao Z. M. (2021). Research on the influencing factors of differentiated academic performance of undergraduates in high-level universities in China. J. High. Educ. 10, 79–87.

[ref7] Buie D. D. (2018). Beyond a deficit view: Understanding the experiences of first-generation students who participate in college access and success community-based organizations Aurora University. Ann Arbor, MI, United States: ProQuest Dissertations Publishing.

[ref8] Chang J. Wang S. W. Mancini C. McGrath-Mahrer B. Orama de Jesus S. (2020). The complexity of cultural mismatch in higher education: norms affecting first-generation college students' coping and help-seeking behaviors. Cult. Divers. Ethn. Minor. Psychol. 26, 280–294. doi: 10.1037/cdp0000311, PMID: 31613122

[ref9] Chen M. Kang Y. J. (2018). From the farm into the elite University of Young People: "sensible" and unintended consequences. China Youth Study 5, 68–75. doi: 10.19633/j.cnki.11-2579/d.2018.0076

[ref10] Covarrubias R. Romero A. Trivelli M. (2015). Family achievement guilt and mental well-being of college students. J. Child Fam. Stud. 24, 2031–2037. doi: 10.1007/s10826-014-0003-8

[ref11] Curtin T. R. Cahalan M. W. (2005). A profile of the veterans upward bound program: 2000–2001. US Department of Education. Washington, DC

[ref12] Dumais S. A. Ward A. (2010). Cultural capital and first-generation college success. Poetics 38, 245–265. doi: 10.1016/j.poetic.2009.11.011

[ref13] Evans N. J. Forney D. S. Guido F. M. Patton L. D. Renn K. A. (2009). Student development in college: Theory, research, and practice. John Wiley & Sons. San Francisco

[ref14] Froiland J. M. Worrell F. C. (2016). Intrinsic motivation, learning goals, engagement, and achievement in a diverse high school. Psychol. Sch. 53, 321–336. doi: 10.1002/pits.21901

[ref15] Garriott P. O. (2020). A critical cultural wealth model of first-generation and economically marginalized college students' academic and career development. J. Career Dev. 47, 80–95. doi: 10.1177/0894845319826266

[ref16] Gibbons M. M. Woodside M. (2014). Addressing the needs of first-generation college students: lessons learned from adults from low-education families. J. Coll. Couns. 17, 21–36. doi: 10.1002/j.2161-1882.2014.00045.x

[ref17] Glass L. E. (2023). Social capital and first-generation college students: examining the relationship between mentoring and college enrollment. Educ. Urban Soc. 55, 143–174. doi: 10.1177/00131245221076097

[ref18] Guo F. Zhao L. Lian Z. X. (2018). Reliability of self-reported data in college student engagement surveys: social desirability Bias in self-reported survey. J. East China Norm. Univ. 36, 53–61+163. doi: 10.16382/j.cnki.1000-5560.2018.04.005

[ref19] Gutierrez-Serrano G. Romo L. F. Chagolla D. (2022). Latina first-generation college students’ motivation to persist: an attribution theory and self-determination theory perspective. J. Lat. Educ. 22, 1–14. doi: 10.1080/15348431.2022.2096611

[ref20] Hagenauer G. Muehlbacher F. Ivanova M. (2023). “It’s where learning and teaching begins–is this relationship”—insights on the teacher-student relationship at university from the teachers’ perspective. High. Educ. 85, 819–835. doi: 10.1007/s10734-022-00867-z, PMID: 37128236 PMC10140077

[ref21] Harackiewicz J. M. Priniski S. J. (2018). Improving student outcomes in higher education: the science of targeted intervention. Annu. Rev. Psychol. 69, 409–435. doi: 10.1146/annurev-psych-122216-011725, PMID: 28934586 PMC6211287

[ref22] Hébert T. P. (2018). An examination of high-achieving first-generation college students from low-income backgrounds. Gift Child Quart. 62, 96–110. doi: 10.1177/0016986217738051

[ref23] Ives J. Castillo-Montoya M. (2020). First-generation college students as academic learners: a systematic review. Rev. Educ. Res. 90, 139–178. doi: 10.3102/0034654319899707

[ref24] Kitchen J. A. Perez R. Hallett R. Kezar A. Reason R. (2021). Ecological validation model of student success: a new student support model for promoting college success among low-income, first-generation, and racially minoritized students. J. Coll. Stud. Dev. 62, 627–642. doi: 10.1353/csd.2021.0062

[ref25] Kuh G. D. (2009). What student affairs professionals need to know about student engagement. J. Coll. Stud. Dev. 50, 683–706. doi: 10.1353/csd.0.0099

[ref26] Kuh G. D. Hu S. (2001). Learning productivity at research universities. J. High. Educ. 72, 1–28. doi: 10.2307/2649131

[ref27] LeBouef S. (2023). Pivoting from deficit to wealth: The role of familial support for first-generation college students (Doctoral dissertation, University of Minnesota). Ann Arbor, MI, United States: ProQuest Dissertations Publishing.

[ref28] LeBouef S. Dworkin J. (2021). First-generation college students and family support: a critical review of empirical research literature. Educ. Sci. 11:294. doi: 10.3390/educsci11060294

[ref29] López M. J. Santelices M. V. Taveras C. M. (2023). Academic performance and adjustment of first-generation students to higher education: a systematic review. Cogent Educ. 10:2209484. doi: 10.1080/2331186X.2023.2209484

[ref30] Manzoni A. Streib J. (2019). The equalizing power of a college degree for first-generation college students: disparities across institutions, majors, and achievement levels. Res. High. Educ. 60, 577–605. doi: 10.1007/s11162-018-9523-1

[ref31] Marco-Bujosa L. M. Baker L. Malott K. M. (2023). “Why am I here?”: a phenomenological exploration of first-generation college student experiences in STEM majors within a predominantly white institution. J. Res. Sci. Teach. 90:003465431989970. doi: 10.1002/tea.21911

[ref32] McFadden D. L. (2016). Health and academic success: a look at the challenges of first-generation community college students. J. Am. Assoc. Nurse Pract. 28, 227–232. doi: 10.1002/2327-6924.12345, PMID: 26960152

[ref33] Mitchall A. M. Jaeger A. J. (2018). Parental influences on low-income, first-generation students’ motivation on the path to college. J. High. Educ. 89, 582–609. doi: 10.1080/00221546.2018.1437664

[ref34] Museus S. D. Chang T. H. (2021). The impact of campus environments on sense of belonging for first-generation college students. J. Coll. Stud. Dev. 62, 367–372. doi: 10.1353/csd.2021.0039

[ref35] NASPA. (2020). National Data Fact Sheets On First-Generation College Students. Available at: https://firstgen.naspa.org/journal-and-research/national-data-fact-sheets-on-first-generation-college-students/7A515490-E6AA-11E9-BAEC0242AC100002

[ref36] Page L. C. Kehoe S. S. Castleman B. L. Sahadewo G. A. (2019). More than dollars for scholars: the impact of the Dell scholars program on college access, persistence, and degree attainment. J. Hum. Resour. 54, 683–725. doi: 10.3368/jhr.54.3.0516.7935R1

[ref37] Pascarella E. T. Pierson C. T. Wolniak G. C. Terenzini P. T. (2004). First-generation college students: additional evidence on college experiences and outcomes. J. High. Educ. 75, 249–284. doi: 10.1080/00221546.2004.11772256

[ref38] Phillips L. T. Stephens N. M. Townsend S. S. Goudeau S. (2020). Access is not enough: cultural mismatch persists to limit first-generation students’ opportunities for achievement throughout college. J. Pers. Soc. Psychol. 119, 1112–1131. doi: 10.1037/pspi0000234 32105102

[ref39] Pratt I. S. Harwood H. B. Cavazos J. T. Ditzfeld C. P. (2019). Should I stay or should I go? Retention in first-generation college students. J. Coll. Stud. Retent.: Res., Theory Prac. 21, 105–118. doi: 10.1177/1521025117690868

[ref40] Riehl R. J. (1994). The academic preparation, aspirations, and first-year performance of first-generation students. College Univ. 70, 14–19.

[ref41] Rocconi L. M. Liu X. Pike G. R. (2020). The impact of person-environment fit on grades, perceived gains, and satisfaction: an application of Holland’s theory. High. Educ. 80, 857–874. doi: 10.1007/s10734-020-00519-0

[ref42] Roksa J. Kinsley P. (2019). The role of family support in facilitating academic success of low-income students. Res. High. Educ. 60, 415–436. doi: 10.1007/s11162-018-9517-z

[ref43] Rubin M. (2012). Social class differences in social integration among students in higher education: a meta-analysis and recommendations for future research. J. Divers. High. Educ. 5, 22–38. doi: 10.1037/a0026162

[ref45] Stephens N. M. Townsend S. S. Dittmann A. G. (2019). Social-class disparities in higher education and professional workplaces: the role of cultural mismatch. Curr. Dir. Psychol. Sci. 28, 67–73. doi: 10.1177/0963721418806506

[ref46] Tao Y. Meng Y. Gao Z. Yang X. (2022). Perceived teacher support, student engagement, and academic achievement: a meta-analysis. Educ. Psychol. 42, 401–420. doi: 10.1080/01443410.2022.2033168

[ref47] Tian J. Yu X. L. (2021). From deficit perspective to advantage perspective: a literature review on first generation college students. Chongqing High. Educ. Res. 5, 106–118. doi: 10.15998/j.cnki.issn1673-8012.2021.05.010

[ref48] Wang T. R. Nuru A. K. (2017). “He wanted me to achieve that for our family and I did, too”: exploring first-generation students’ experiences of turning points during the transition to college. J. Fam. Commun. 17, 153–168. doi: 10.1080/15267431.2016.1264401

[ref49] Watts G. W. Garfield T. A. Davis M. T. (2023). Experiences, supports, and strategies of first-generation college students. Coll. Teach. 71, 38–48. doi: 10.1080/87567555.2022.2050669

[ref50] Williams S. M. Ferrari J. R. (2015). Identification among first-generation citizen students and first-generation college students: an exploration of school sense of community. J. Community Psychol. 43, 377–387. doi: 10.1002/jcop.21685

[ref51] Yu X. L. Han Y. (2018). How to cultivate successful people from poor families—on the breakthrough of class restrictions from the cultural capital perspective. J. High. Educ. 2, 8–16.

[ref52] Zhang H. F. Guo F. Shi J. H. (2017). On improving first-generation college Students' participation in high-impact educational practices. Educ. Res. 6, 32–43.

[ref53] Zhang H. F. Shi J. H. Zhou X. T. (2021). A study on China college Students' learning motivation in the early era of higher education universalization. Tsinghua J.Educ. 4, 141–148. doi: 10.14138/j.1001-4519.2021.04.014108

[ref54] Zhang H. F. Zhao L. Guo F. (2016). A portrait of first-generation college students in China: an analysis based on the China college student survey. Tsinghua J. Educ. 37:72-78+94. doi: 10.14138/j.1001-4519.2016.06.007207

[ref55] Zhao L. Wang W. Li Y. F. Ji H. C. Shi J. H. (2014). A study on the mechanism of the influence of pre-college experience on higher education quality with an analysis of the comprehensive reforms in the field of education. Tsinghua J.Educ. 3, 35–44. doi: 10.14138/j.1001-4519.2014.03.020

